# Molecular pattern of a decrease in the rewarding effect of cocaine after an escalating-dose drug regimen

**DOI:** 10.1007/s43440-022-00443-3

**Published:** 2022-12-31

**Authors:** Karolina Kołosowska, Małgorzata Lehner, Anna Skórzewska, Aleksandra Gawryluk, Filip Tomczuk, Alicja Sobolewska, Danuta Turzyńska, Monika Liguz-Lęcznar, Małgorzata Bednarska-Makaruk, Piotr Maciejak, Aleksandra Wisłowska-Stanek

**Affiliations:** 1 Department of Neurochemistry, Institute of Psychiatry and Neurology, 9 Sobieskiego Street, 02-957 Warsaw, Poland; 2 Department of Molecular and Cellular Neurobiology, Nencki Institute of Experimental Biology, Polish Academy of Sciences, 3 Pasteur Street, 02-093 Warsaw, Poland; 3 Department of Genetics, Institute of Psychiatry and Neurology, 9 Sobieskiego Street, 02-957 Warsaw, Poland; 4 Department of Experimental and Clinical Pharmacology, Medical University of Warsaw, Centre for Preclinical Research and Technology (CePT), 1B Banacha Street, 02-097 Warsaw, Poland

**Keywords:** Cocaine binge, HPA axis, Neuromodulators, KOR, OX1R, microRNA

## Abstract

**Background:**

Long-term cocaine exposure leads to dysregulation of the reward system and initiates processes that ultimately weaken its rewarding effects. Here, we studied the influence of an escalating-dose cocaine regimen on drug-associated appetitive behavior after a withdrawal period, along with corresponding molecular changes in plasma and the prefrontal cortex (PFC).

**Methods:**

We applied a 5 day escalating-dose cocaine regimen in rats. We assessed anxiety-like behavior at the beginning of the withdrawal period in the elevated plus maze (EPM) test. The reinforcement properties of cocaine were evaluated in the Conditioned Place Preference (CPP) test along with ultrasonic vocalization (USV) in the appetitive range in a drug-associated context. We assessed corticosterone, proopiomelanocortin (POMC), β-endorphin, CART 55–102 levels in plasma (by ELISA), along with mRNA levels for D2 dopaminergic receptor (D2R), κ-receptor (KOR), orexin 1 receptor (OX1R), CART 55–102, and potential markers of cocaine abuse: miRNA-124 and miRNA-137 levels in the PFC (by PCR).

**Results:**

Rats subjected to the escalating-dose cocaine binge regimen spent less time in the cocaine-paired compartment, and presented a lower number of appetitive USV episodes. These changes were accompanied by a decrease in corticosterone and CART levels, an increase in POMC and β-endorphin levels in plasma, and an increase in the mRNA for D2R and miRNA-124 levels, but a decrease in the mRNA levels for KOR, OX1R, and CART 55–102 in the PFC.

**Conclusions:**

The presented data reflect a part of a bigger picture of a multilevel interplay between neurotransmitter systems and neuromodulators underlying processes associated with cocaine abuse.

**Supplementary Information:**

The online version contains supplementary material available at 10.1007/s43440-022-00443-3.

## Introduction

A common symptom in cocaine addicts is impairment of reward anticipation. We know so far that long-term dose-escalating psychostimulant (cocaine) administration leads to dysregulation of the reward system and initiates processes that ultimately result in the weakening of the dopaminergic signal reflected by a decrease in the rewarding effect of a drug [1–7]. This phenomenon may lead to increased drug consumption, compulsive drug-seeking, and loss of control over drug use despite its adverse consequences [8, 9].

Cortical regions are implicated in the control of reward-seeking behavior and reinforcement mechanisms associated with cue-potentiated behavior [10–16]. The bidirectional regulation occurs between the ventral tegmental area (VTA) dopaminergic neurons and its target, the medial prefrontal cortex (mPFC), playing an essential role in control over reward system activity [9, 17, 18].

Dysregulation of the hypothalamic–pituitary–adrenal (HPA) axis is also supposed to contribute to craving and early relapse-associated processes [19, 20]. A lot of research points to the role of neuromodulators related to the HPA axis and hypothalamic peptides, the cocaine and amphetamine-regulated transcript (CART 55–102), and orexins, which are highly expressed in structures composing the HPA axis, as modulators of dopaminergic neurotransmission [12–14, 16, 21–23]. Orexin neurons co-express the inhibitory opioid dynorphin, and those two peptides are characterized by opposing actions on motivated behavior, namely, orexin is implicated in states of arousal and reward, whereas dynorphin is implicated in depressive-like states [24]. Orexin signaling in the PFC is implicated in the reinstatement of reward seeking [25–28].

In the current study, we were looking for a molecular pattern associated with an altered appetitive response to cocaine after the withdrawal period following dose-escalating administration. In humans, cocaine is usually consumed in recurrent cycles, which occur over 1–7 days with a period of abstinence accompanied by depressive symptoms and anxiety [29–35]. In the current study, we applied an escalating-dose cocaine regimen in rats, which was modeled to mimic the human pattern of cocaine abuse. In the study, we assessed anxiety-like behavior in the early withdrawal period in the Elevated Plus Maze (EPM) test, as anxiety and depression-related behavior prevails typically during the initial period of abstinence [36]. The reinforcement properties of cocaine were evaluated in the Conditioned Place Preference (CPP) test [37, 38] along with ultrasonic vocalization (USV) in the appetitive range (30–120 kHz, mainly referred to as 50 kHz) in the drug-associated context, often used to measure appetitive response to the context of various rewarding stimuli [39].

In search for a molecular pattern associated with altered response to cocaine we measured neuromodulators related to the HPA axis and hypothalamic peptides, namely corticosterone, proopiomelanocortin (POMC), β-endorphin, along with CART 55–102 levels in plasma, and mRNA levels for D2 dopaminergic receptor, κ-opioid receptor (KOR), orexin 1 receptor (OX1R), CART 55–102, and previously suggested markers of cocaine abuse: microRNA-124 (miRNA-124) and microRNA-137 (miRNA-137) levels, in the PFC [40–42].

## Materials and methods

### Animals

Male Wistar adult rats (*n* = 20), 9-week old and weighing 220–250 g at the beginning of the experiment, purchased from a licensed breeder (The Center for Experimental Medicine of the Medical University, 24 A Skłodowskiej-Curie Street, Białystok, Poland) were used in the study. The animals were housed in environmentally enriched laboratory conditions (temperature 20 ± 2 °C; 12 h light/dark cycle, light on at 7 am; 45–55% humidity, the cages were enriched with wood for gnawing). The rats were housed per 4 in opaque plastic cages (55 × 33 cm floor size, *H* = 19.5 cm) with free access to standard laboratory rat chow and tap water.

The study was conducted following the European Communities Council Directive 2010/63/UE. The Local Committee for Animal Care and Use at the Medical University of Warsaw, Poland, approved this study (Protocol No. WAW2/073/2018). All care was administered to minimize animal discomfort during experimental procedures. We confirm that the animals did not suffer unnecessarily at any stage of the experiments.

### Experimental scheme (Fig. 1)

**Fig. 1:**
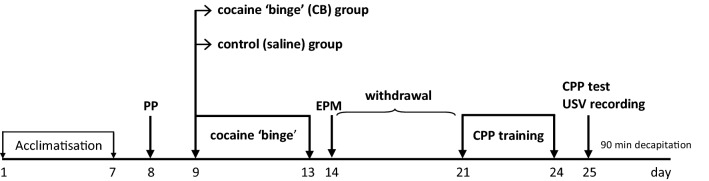
The experimental scheme. *PP* natural place preference assessment, *EPM* elevated plus maze test, *CPP* condition place preference, *USV* ultrasonic vocalization

After 7 days of acclimatization to the vivarium, the natural place preference of the animals was assessed. Next, the cocaine ‘binge’ regimen was introduced for 5 days. Twenty-four hours after the last cocaine injection, the EPM test was performed, and a withdrawal procedure was carried out for 7 days. Subsequently, the rats were trained for the CPP for 4 days. Two rats were excluded from the experiments for technical reasons. The day after, the place preference in the cocaine-paired compartment was assessed, and 50-kHz USVs were recorded simultaneously. Ninety minutes later, animals were decapitated.

### Behavioral assessment

#### Escalating dose ‘binge’ cocaine regimen

The escalating-dose cocaine regimen consisted of 15 cocaine injections at increasing doses. Cocaine hydrochloride (TRC, Canada) was dissolved in a sterile aqueous 0.9% NaCl solution (Polpharma, Starogard Gdański, Poland) and injected intraperitoneally. Half of the animals (cocaine ‘binge’, CB group, *n* = 10) were injected with cocaine three times a day (at 7:45 am; 9:45 am; and 11:45 am) for 5 consecutive days in the following scheme: 1st day—three injections, 10 mg/kg, 2nd day—three injections, 15 mg/kg; 3rd day—two injections, 20 mg/kg (an adjusted volume 2 ml/kg) and one injection, 25 mg/kg; 4th day—two injections, 25 mg/kg and one injection, 30 mg/kg; 5th day—three injections, 30 mg/kg. Other animals (control group, *n* = 10) were given saline (sterile aqueous 0.9% NaCl solution) in the same regimen. The cocaine and saline injections were administered in dark cages (55 × 33 cm floor size, *H* = 19 cm) with no bedding for 40 min.

#### EPM test

The EPM apparatus was made of wood and consisted of two opposed open arms, two opposed walled arms (arm floor sizes, 50 × 10 cm), and an open square (10 × 10 cm) in the center, as it was described in our previous study [43]. The rats were transferred individually to the testing room, placed on the central square, facing an open arm, and had 5 min access to explore the maze. The behavior was recorded with a ceiling-mounted EV-650CG video camera (Sony, Japan) connected to a PC equipped with the EthoVision XT VideoTracking System v.7 (Noldus Information Technology B.V., Wageningen, The Netherlands). Video recordings were then used to calculate the total time spent in the open arms by an independent observer.

#### CPP procedure

The apparatus consisted of a plywood box (with 34 cm high walls) divided into two main compartments (35.5 × 20 cm floor size) separated by a smaller compartment (10 × 20 cm floor size). The apparatus and procedure were previously described by Taracha et al., 2014 [44]. Two CPP apparatuses were used simultaneously, thoroughly cleaned after each rat. The apparatuses were separated with a 1.1 × 0.7 m (L × H) sound-attenuating wall made of a 2-cm thick particle board with a black veneer on both sides. Rats’ behaviors in non-preferred and preferred areas were recorded with a ceiling-mounted EV-650CG video camera (Sony, Japan) and analyzed by the computerised system EthoVision XT VideoTracking System v.7 (Noldus Information Technology B.V., Wageningen, The Netherlands). Natural place preference was determined in a 15 min pre-test at the beginning of the experiment. The CCP training was carried out for 4 consecutive days. On the first day, the rats were given an i.p. saline injection and were instantly confined to the preferred section of the apparatus for 40 min. On the next day, designated rats received a cocaine injection and were immediately confined to the non-preferred section of the apparatus for 40 min. The 10 mg/kg cocaine (an adjusted volume 2 ml/kg) dose was used in all CPP training sessions. The training procedure was repeated over the next two days. The cocaine injections were performed between 7:45 am and 11:45 am. On the 5th day, all the rats were given access to the open CPP apparatus for 20 min (of which the first 15 min were for CPP analysis) to examine place preference and USVs response. The training sessions (conditioning) and the testing were performed at the same time of day. The CPP was calculated as the difference between the times spent in the drug-paired compartment during the test and pre-test [44, 45].

#### 50-kHz USV recording

The USVs were recorded during the CPP test session. The USVs were recorded with a single CM16 condenser microphone (Avisoft Bioacoustics, Berlin, Germany) placed face down 35 cm above the testing box floor and centrally above the rat-accessible area. The microphone was sensitive to frequencies of 15–180 kHz, had a flat response characteristic (± 6 dB) within the 25–140 kHz frequency range and was connected to a custom-made amplifier of 600 Ω input impedance, 16 v/v (12 dB) voltage gain, and ± 0.1 dB (30 Hz–100 kHz) frequency response. The amplified signal was passed to an adjacent (observer-occupied) room, processed with a custom-made antialiasing filter, and then sent to a PC equipped with a model PCI-703-16A (Eagle Technology, Eagle River, WI, USA) acquisition board (14-bit, 400 kHz) and custom-written software (Rat-Rec Pro 5.0), as previously described [45]. The sound proofing method was validated [45, 46]. The recorded data were processed using the RAT-REC PRO 5.0 software (custom-made, Warsaw, Poland) and displayed as colour spectrograms. We did not observe the 22-kHz calls. Frequency-modulated (FM) and non-FM (‘flat’) 50-kHz calls were identified using the characteristics specified in earlier studies by an independent observer. Each 50-kHz signal was manually marked by the section label to be included in the automatic calls’ counting [44–46].

### Biochemical and molecular analysis

#### Tissue preparation for ELISA and real-time PCR

After decapitation, the trunk blood samples were taken and stored at − 20 °C for ELISA assay, while the brains were removed. The PFC (4.7 to 4.2 anterior to bregma), designated for mRNA assessment, was crudely dissected from the brain. The obtained tissue was placed in stayRNA (A@A Biotechnology, Poland), frozen and stored at − 70 °C for further analyses.

#### ELISA analyses

##### Plasma corticosterone levels

Plasma corticosterone concentrations were analyzed by Corticosterone rat/mouse ELISA kit (Demeditec Diagnostics GmbH., Germany). We used 10 μl plasma per well for the assay. The sensitivity of the corticosterone assay was 11.83 nmol/l. The corticosterone antibody cross-reactivity with other naturally occurring adrenal steroids was not detectable, except for 11-deoxycorticosterone (2.4%), progesterone (0.7%), cortisol (0.3%), and aldosterone (0.2%). The inter-and intra- assay coefficients of variance were 8.2 and 5.3%, respectively [47].

##### Plasma CART levels

Plasma CART concentrations were analyzed by CART Enzyme Immunoassay Kit, RayBiotech, sensitivity (minimum detectable concentration was 7.2 pg/ml, Standard curve range was 1–10.000 ng/ml). We used 10 μl of plasma per well, for the assay. All samples were twofold diluted as recommended by the kit manufacturer; the dilution factor was included in the analysis software. The inter-intra assay coefficients of variance were < 15% and 10%, respectively.

##### Plasma β-endorphin levels

Plasma β-endorphin concentrations were analyzed by Rat Beta-Endorphin, SunRed, sensitivity 3. 127 ng/L, Assay range was 5 ng/L–900 ng/L. We used 10 μl of plasma per well, volume-dissolved 1:5 in PBS for the assay (according to the previous examination).

##### Plasma POMC levels

Plasma POMC concentrations were analyzed by Rat for Proopiomelanocortin, BioSource, sensitivity 33 pg/mL, Detection (Assay) 78–5.000 pg/mL. We used 10 μl of plasma per well, volume-dissolved 1:2 in PBS for the assay, according to the prescription. The inter-and intra assay coefficients of variance were < 12 and 10%, respectively.

#### Real-time PCR

The total RNA was extracted using the miRNeasy Mini Kit (Qiagen, Germany), according to the manufacturer’s instructions. The concentration and purity of total RNA were determined using spectrophotometry (NanoDrop 2000/2000c, Thermo Scientific, USA). All samples had Abs 260/280 > 1.9 and 260/230 > 1.4.

Reverse transcription of mRNA was performed using the RevertAid First Strand cDNA Synthesis Kit (Thermo Scientific, USA) in a total volume of 20 μl according to the manufacturer’s instructions. Reverse transcription of miRNA was performed using TaqMan^®^ MicroRNA Reverse Transcription Kit and TaqMan^®^ Gene Expression Assays specific primers (Thermo Scientific, USA). Real-time PCR analysis was performed using PikoReal™ Real-Time PCR System (Thermo Fisher Scientific) with PowerSYBR® Green PCR Master Mix (Applied Biosystems). The PCR-specific primers used in this study are as follows: D2 dopamine receptor mRNA (5′ → 3′, F:CAACAATACAGACCAGAATGAG; R:CAGCAGAGTGACGATGAA), CART mRNA (5′ → 3′, F:GATCGGGAAGCTGTGTGACT; R:ATTTTGAAGCAGCAGGGAAA), KOR mRNA (5′ → 3′, F:GGCAGCAAGTGTGAAGAACA; R:GGTGCCCAGTAAGTTTTGGA), OX1R mRNA (5′ → 3′, F:GCGCGATTATCTCTATCCGAA; R:AAGGCTATGAGAAACACGGCC). Housekeeping reference genes: glyceraldehyde-3-phosphate dehydrogenase (GAPDH, 5′ → 3′, F:ATGACAATGAATATGGCTACA; R:CTCTTGCTCTCAGTATCCTT) and peptidylprolyl isomerase A (PPIA, 5′ → 3′, F:AATGGCACTGGTGGCAAGTC; R:GCCAGGACCTGTATGCTTCAG. cDNA (concentration 5 ng/μl) was amplified for each sample in a total volume of 10 μl.

The amplification reaction included 40 cycles with a 95 °C denaturation step for 5 s and a 61 °C annealing step for 45 s and was preceded by 95 °C initial denaturation for 1 min. A dissociation stage was performed to assess the specificity of primers. Each sample was run in a triplicate. Real-time PCR assays of total RNA were performed to measure the expression levels of miRNA-124 and miRNA-137. Relative levels were normalized to U6 snRNA (Control Sequence: GTGCTCGCTTCGGCAGCACATATACTA AAATTGGAAC GATACAGAGAAGATTAGCATGGCCCCTGCGCAAGGAT GACACGCA ATTCGTGAAGCGTTCCATATTTT) and 4.5S RNA(H) (Control Sequence: GCCGGTTGTGGTGGCGCACACCGGTAGGATTTGC TGAAGGAGGCAGAG GCAGGAGGATCACGAGTTCGAGGCCAGCCTGG GCTACACATTT).

Analysis of miRNAs was also performed using PikoReal™ Real-Time PCR System (Thermo Fisher Scientific, USA) with TaqMan^®^ Universal Master Mix II, no UNG(Applied Biosystems), specific TaqMan^®^ Probes (TaqMan™ miRNA Assays, Termofisher Scientific, in Table 1) and products of reverse transcription. Each sample was run in triplicate. Analysis of all real-time PCR data was performed using the comparative ΔΔCT method [48].

**Table 1:** TaqMan^TM^ MicroRNA Assays: mmu-miR-124a, hsa-miR-137

Assay name	miRBase ID	miRBase accession numbers	Mature miRNA sequence
mmu-miR-124a	mmu-miR-124-3p	MIMAT0000134	UAAGGCACGCGGUGAAUGCC
hsa-miR-137	hsa-miR-137-3p	MIMAT0000429	UUAUUGCUUAAGAAUACGCGUAG

### Statistical analysis

The statistical analyses were performed using Statistica v.12 software. We used the Shapiro–Wilk test to assess the data distribution. Some variables presented a normal distribution, namely the time spent in the open arms in the EPM test, the total distance in the CPP test, β-endorphin levels, miRNA-124, and miRNA-137 levels in the PFC. We performed statistical analysis using the Student’s *t* test for variables with normal distribution. The data sets that did not present the normal distribution were analyzed with the Mann–Whitney *U* test. The data are presented as the mean + the standard error of the mean (SEM).

## Results

### Behavioral analysis: EPM test and CPP test (Fig. 2)

**Fig. 2:**
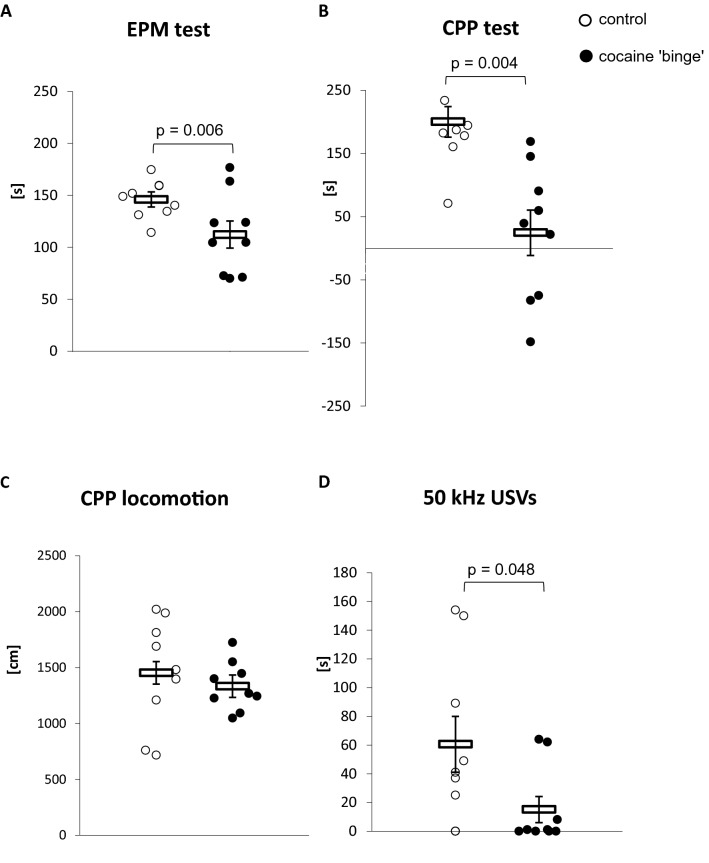
**A** Time spent in open arms in the EPM test after cocaine ‘binge’; **B** Shift of time spent in the cocaine-associated compartment during the CPP test; **C** Locomotor activity in the CPP test; **D** 50-kHz USVs in the cocaine-associated context during the CPP test. *CPP* conditioned place preference test, *EPM* elevated plus maze test. The time spent in the open arms in the EPM test and the total distance in the CPP test were analyzed with the Student *t* test. The shift of time spent in cocaine-associated compartment during the CPP test and the number of 50-kHz USVs in the cocaine-associated context during the CPP test were analyzed by the Mann–Whitney *U* test. Control-group receiving saline (*n* = 7–9), cocaine ‘binge’–cocaine ‘binge’ group (*n* = 9). The data are shown as the means + SEM

The CB rats spent significantly less time in the open arms in the EPM test compared to the control group (*t*_16_ = 3.12, *p* = 0.006), whereas no differences in the locomotor activity measured as the total distance crossed during the test were observed between groups (*U* = 37, N1 = 9, N2 = 9, *p* = 0.791).

Mann–Whitney *U* test revealed that the CB rats spent significantly less time in the cocaine-paired compartment (*U* = 4.0, N1 = 7, N2 = 9, *p* = 0.004) and demonstrated less episodes of 50-kHz USV compared to the control rats (*U* = 15.0, N1 = 8, N2 = 9, *p* = 0.048). No significant differences between the experimental groups were found in the total distance crossed in the CPP test (*t*_14_ = 0.86, *p* = 0.401).

### Corticosterone, POMC, CART 55–102, and β-endorphin levels in the plasma (Fig. 3)

**Fig. 3:**
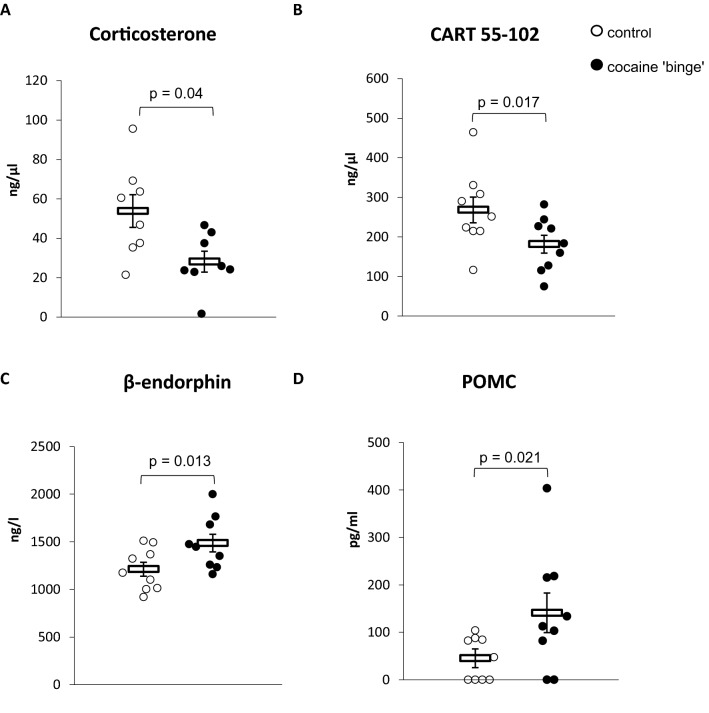
**A** Corticosterone; **B** CART 55–102; **C** β-endorphin; **D** POMC levels in the plasma. CART—cocaine amphetamine regulated transcript 55–102; POMC—proopiomelanocortin peptide. The β-endorphin levels were analyzed with the Student *t* test. The corticosterone, CART 55–102, and POMC levels were analyzed by the Mann–Whitney *U* test. Control-group receiving saline (*n* = 8–9), cocaine ‘binge’–cocaine ‘binge’ group (*n* = 8–9). The data are shown as the means + SEM

The CB rats showed significantly lower plasma corticosterone concentrations (*U* = 12, N1 = 8, N2 = 8, *p* = 0.04) and CART 55–102 levels (*U* = 13.0, N1 = 9, N2 = 9, *p* = 0.017), while plasma concentrations of β-endorphin (*t*_16_ = -2.8, *p* = 0.013) and POMC (*U* = 14, N1 = 9, N2 = 9, *p* = 0.021) were significantly higher when compared to the control group.

### mRNA levels for D2R, OX1R, KOR, and CART 55–102 in the PFC (Fig. 4)

**Fig. 4:**
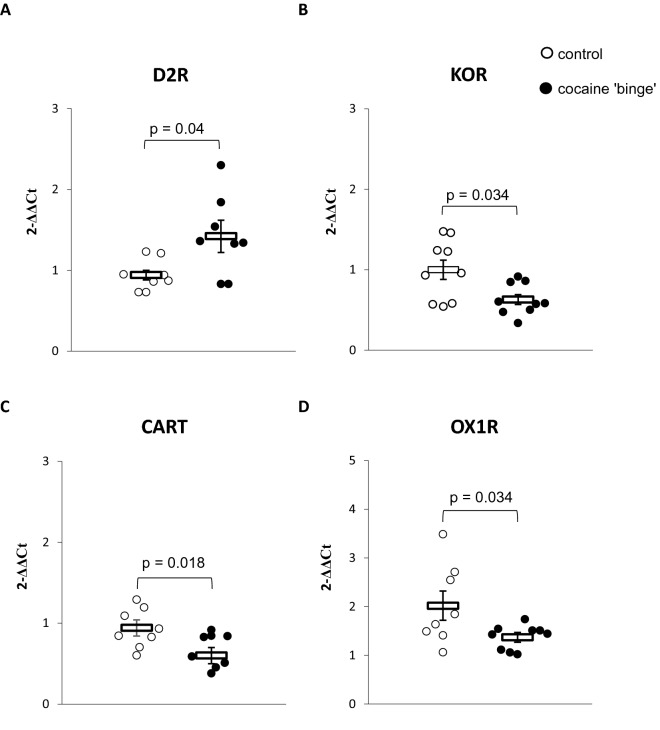
mRNA levels in the prefrontal cortex (PFC) for **A** Dopamine type 2 receptor (D2R); **B** Kappa opioid receptor (KOR); **C** Cocaine and amphetamine-regulated transcript 55–102 (CART 55–102); **D** Orexin type 1 receptor (OX1R). The presented data were analyzed by the Mann–Whitney *U* test. Control-group receiving saline (*n* = 8–9), cocaine ‘binge’–cocaine ‘binge’ group (*n* = 8–9). The data are shown as the means + SEM

Mann–Whitney *U* test revealed that the CB rats presented higher D2R mRNA (*U* = 12.0, N1 = 8, N2 = 8, *p* = 0.04), lower OX1R mRNA (*U* = 11, N1 = 8, N2 = 9, *p* = 0.018), lower CART 55–102 mRNA (*U* = 13.5, N1 = 8, N2 = 9, *p* = 0.034), and lower KOR mRNA levels (*U* = 16, N1 = 9, N2 = 9, *p* = 0.034), compared to the control rats.

### miRNA-124 and miRNA-137 levels in the PFC (Fig. 5)

**Fig. 5:**
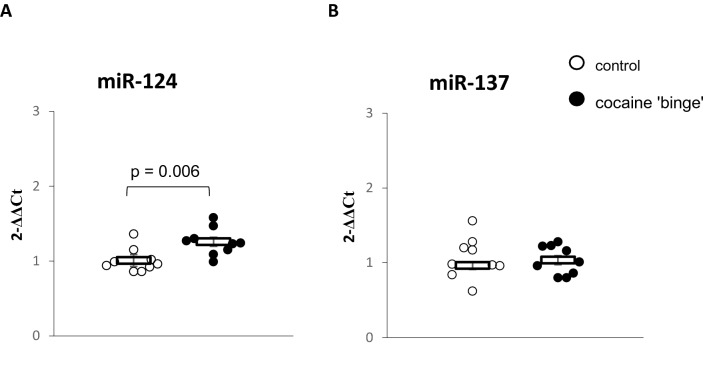
**A** miRNA-124 and **B** miRNA-137 levels in the prefrontal cortex (PFC). The presented data were analyzed with Student t-test. Control-group receiving saline (*n* = 9), cocaine ‘binge’–cocaine ‘binge’ group (*n* = 9). The data are shown as the means + SEM

The CB rats presented higher miRNA-124 levels compared to the control rats (*t*_16_ = 3.12, *p* = 0.006). No differences were observed between the groups in the miRNA-137 levels (*t*_16_ = 0.26, *p* = 0.8).

## Discussion

In our study, we demonstrated that the CB rats exposed to an escalating-dose cocaine regimen showed enhanced anxiety at the beginning of the withdrawal period defined as a decrease in the number of visits in the open arms in the EPM test, and diminished cocaine-associated appetitive response after the withdrawal period measured as less time spent in the cocaine-paired compartment and a lower number of appetitive USV episodes. The changes in appetitive behavior were accompanied by molecular alterations in plasma and the PFC. Specifically, we observed a decrease in corticosterone and CART 55–102 levels, and an increase in β-endorphin and POMC levels in the plasma of the CB rats, along with an increase in the mRNA for D2R, but a decrease in the mRNA levels for OX1R, KOR, and CART55-102 in the PFC. We also found an increase in the miRNA-124 level in the PFC of the CB rats.

At the beginning of the withdrawal period that followed cocaine binge, we observed enhanced anxiety-like behavior in the CB rats, similarly to other studies [49, 50]. Also in human cocaine abusers, discontinuation of drug intake usually produces a variety of adverse withdrawal symptoms among which anxiety and depression-related behaviors are prevailing during the initial period of abstinence [36]. Moreover, the CB rats showed a weaker place preference following cocaine’s 7 day withdrawal period, most possibly because of an increase in reward threshold [51]. In other studies, the rewarding effect of cocaine was reported to have been restored 14 days after cocaine withdrawal [38]. The sensitization process is observed in the early period of cocaine use, while tolerance appears over time of administration, phenomena established in both rodents and humans [52, 53]. Although it is a passive paradigm of cocaine administration compared to self-administration, the escalating dose regimen seems to at least partly mimic the cocaine binge, namely, the increase in cocaine intake. The studies of Calipari et al. [54, 55] regarding differences in dopamine signaling in different patterns of cocaine self-administration, suggest that tolerance depends not on the pattern of administration, but on the total cocaine intake within sessions. Similar patterns of behavior were demonstrated in our previous study regarding amphetamine self-administration [56].

In our study, we observed lower corticosterone concentration accompanied by an increase in POMC and β-endorphin levels in the plasma of the CB rats. The escalating-dose cocaine administration has been proved to lead to dysregulation of the HPA axis [57–59]. An increase in plasma corticosterone level was observed at the beginning of the chronic binge cocaine administration, while it was reduced on the 14th day of the applied regimen followed by a return to its basal level after 10 days of the withdrawal period [57]. In the study by García-Fuster et al. [59] corticosterone levels progressively decreased during the course of withdrawal from extended daily access of cocaine self-administration, and normalized following 28 days of withdrawal. An enhanced secretion of corticotropin-releasing factor (CRF) and adrenocorticotropic hormone (ACTH), and decreased cortisol levels were observed during the withdrawal period in humans and linked to depression and anxiety symptoms [60, 61]. Here, we can assume that lower corticosterone level may be the result of dysregulation of the HPA axis activity in response to cocaine binge and be related to the diminished cocaine-associated appetitive response after the withdrawal period.

POMC is synthesized in the pituitary gland in the brain and in several peripheral tissues. During posttranslational processing, it can be tissue-specifically cleaved to hormones and neuropeptides with very different biological activities [62]. In the anterior pituitary, POMC is processed predominantly to ACTH, β-lipotropin (β-LPH), and to β-endorphin to a lesser extent [62]. In the study by Zhou and Kreek [63], the increased hypothalamic POMC expression was persistent during the withdrawal period along with an increase in β-endorphin biosynthesis and release, which authors associated with enhanced cocaine seeking. However, continuously increased levels of β-endorphin in plasma were reported not only in abstinent human cocaine addicts, but also during cocaine binges [64]. Except for the postulated role of β-endorphin in mediating the rewarding or reinforcing effects of cocaine, it is also known to be released in response to physical stress [65, 66]. In normal human subjects a decline in ACTH and cortisol plasma levels was associated with elevated β-endorphin, which suggests feedback loop inhibition of pituitary ACTH release or suppression of hypothalamic CRF release by β-endorphin [67]. This observation may also partially explain our results, namely lower corticosterone concentrations along with an increase in β-endorphin and POMC levels in the plasma of the CB rats. However, in the current experimental design, it is difficult to assess to what extent effects of these two processes, namely cocaine withdrawal and cocaine re-exposure, may somehow overlap.

CART 55–102 is known for its properties to modulate the activity of the mesolimbic dopaminergic pathway and to affect reward-seeking behavior [68, 69]. Furthermore, compelling evidence has shown that CART 55–102 is involved in the HPA axis regulation associated with stress response [70–75]. CART 55–102 stimulates CRF and glucocorticoid secretion, whereas CRF and glucocorticoids increase the transcriptional activity of the CART gene [76–78]. The administration of CART 55–102 upregulates ACTH and corticosterone levels through a CRF-dependent mechanism [73, 74]. In light of these data, decreased CART 55–102 levels in plasma and corresponding decreased CART 55–102 mRNA levels in the PFC may be linked to the HPA axis dysregulation and diminished cocaine-associated appetitive response. In the study of Rakovska et al. [69], CART 55–102 administration was associated with a decrease in dopamine in the mouse nucleus accumbens (NAc) and attenuation of cocaine-induced effects on dopamine release.

Drug-withdrawal after chronic cocaine administration decreases dopamine signaling, in contrast to the positive reinforcement induced by the substance [51]. Diminished dopaminergic signaling after repeated cocaine intake may increase the risk of anxiety and dysphoria and lead to depressive-like behavior [79–81]. In our experiment, we detected a rise in the D2R mRNA in the PFC of the CB rats, similar to the study by Frankowska et al. [82], which presents an increased D2R expression in the PFC in the withdrawal period after cocaine self-administration. These phenomena may reflect adaptive changes in D2R expression in the cortex (probably following lower dopamine levels in the cortical-limbic system after exposure to the binge cocaine paradigm and withdrawal), and can be associated with lower appetitive response to cocaine [31].

The modulatory control of the dynorphin/KOR system over dopamine signaling in mesolimbic areas contributes to the development of negative affective states and changes in the perception of reinforcing and aversive stimuli [83]. Chronic cocaine exposure elevates the neuropeptide dynorphin levels, an endogenous ligand at KOR that suppresses dopamine release in the NAc and elicits negative affective states upon drug withdrawal [84–87]. Infusion of a KOR agonist into the PFC decreased dopamine levels in wild-type mice but not in KOR-knock-out mice (a model of a specific deletion of KOR in dopaminergic neurons), confirming KOR-mediated control of dopaminergic transmission in the PFC [88].

In our study, we observed a decrease in the KOR mRNA in the PFC of the CB rats. Although we are aware of some limitations of this study, namely changes in the level of transcripts were not confirmed at the protein level, we suggest that the reduced KOR mRNA may be associated with the lower appetitive response to cocaine-associated context. This speculation is supported by the study by Wee et al. [89] on the effects of KOR blockade on cocaine seeking and consumption in different animal models of cocaine exposure. The KOR blockade selectively reduced cocaine seeking but not cocaine consumption in animals with a history of extended cocaine administration, but not in animals that self-administered it on a short access procedure [89]. These data link the decreased KOR function with lower appetitive response upon withdrawal from chronic cocaine exposure.

In the current study, we also observed a decrease in the OX1R mRNA in the PFC of the CB rats. Orexin neurons, localized mainly in the lateral hypothalamus and projecting through the cortex and limbic system, initiate arousal states and modulate reward system activity [90]. Much evidence supports the important role of OX1R in the prelimbic cortex in addiction-related states [10, 14, 22, 24, 91–93], specifically, the significance of signaling at OX1R in relapse to cocaine-seeking [94]. The OX1R gene was induced in the PFC by cocaine exposure [95]. Systemic administration of OX1R antagonist attenuated cue-induced reinstatement of extinguished cocaine-seeking [96], but did not attenuate reinstatement of responding induced by a priming injection of cocaine [97]. In animals that underwent abstinence from chronic self-administration, the OX1R antagonism reduced the reinstatement of cocaine-seeking [98]. Based on those data, we can assume that lower OX1R mRNA levels in the PFC may be related to lower appetitive response to cocaine-associated context and decreased cortical activity following the withdrawal period.

Repeated intake of drugs of abuse, such as cocaine, promotes alterations in gene expression that underlie addiction-related processes [99–101]. Several studies have shown that cocaine can influence the activity of small non-coding RNAs, miRNAs, known to regulate gene expression on posttranscriptional level [102–104]. Among them, miRNA-124 is considered as a promising biomarker of cocaine abuse as it was down-regulated in a dopaminergic neuron-like model after acute cocaine exposure [105], and found elevated in the blood of cocaine-addicted women during the withdrawal period [106]. In our study, the CB rats that presented lower appetitive vocalization in the cocaine-associated context after the withdrawal period showed also an increased miRNA-124 level in the PFC. Chandrasekar et al. [107] found that lentiviral vector (LV)-miRNA-124 expression in the NAc attenuated cocaine CPP, whereas silencing miRNAs by corresponding LV-miRNA silencers inversed this effect. We are aware of the pleiotropic effects of miRNAs as they can target many different molecular pathways in cell- and tissue-specific manner, thus further research is needed to confirm the targets of these miRNAs in this context. So far, other studies identified PARP-1 [108] and BDNF [109] as plausible direct targets of miRNA-124 in neuronal cells.

Another promising marker of diseases associated with dopaminergic dysfunction is miRNA-137, which regulates DAT expression at the post-transcriptional level [40]. Addiction-prone rats showed elevated miRNA-137 expression in the CNS after extinction and relapse testing following cocaine self-administration [41]. However, we observed no differences in miRNA-137 between the CB and control rats in our study.

## Conclusions

Our study shows that the escalating-dose cocaine regimen resulted in anxiety-like behavior at the beginning of the withdrawal period and reduced cocaine-associated appetitive response afterwards. This behavioral pattern was accompanied by dysregulation of the HPA axis activity followed by changes in related neuromodulators in the plasma, and alterations in mRNA levels for D2, KOR, OX1R, CART 55–120, and miRNA-124, a postulated marker of cocaine abuse, in the PFC. Our observations are in line to a large extent with results of other studies, thereby confirming the impact of cocaine on the HPA axis activity and systems that mediate reinforcing effects of the drug and related affective states. To conclude, the obtained data reflect a part of a bigger picture of a multilevel interplay between neurotransmitter systems and post-transcriptional regulation of gene expression underlying processes associated with cocaine abuse. However, in-depth characteristics of molecular processes in this model require more detailed “cause-and-effect” exploration.

## Limitations

The major limitation of this study is that the levels of transcripts in the PFC were not confirmed at the protein level, however, the obtained data are generally consistent with those presented by other authors. Moreover, target-specific studies regarding plausible miRNA-regulated molecular pathways associated with cocaine biological effects should be undertaken.

## Supplementary Information

Below is the link to the electronic supplementary material.

Supplementary file1 (DOC 29 kb)

## Data Availability

The datasets generated during and/or analyzed during the current study are available from the corresponding author on reasonable request.

## Abbreviations

ACTH: Adrenocorticotropic hormone
BDNF: Brain-derived neurotrophic factor
CART 55–102: Cocaine and amphetamine-regulated transcript 55–102
CB rats: Cocaine ‘binged’ rats
CNS: Central nervous system
CPP test: Conditioned place preference test
CRF: Corticotropin-releasing factor
D2R: D2 dopaminergic receptor
DAT: Dopamine active transporter
ELISA: Enzyme-linked immunosorbent assay
EPM test: Elevated plus maze test
HPA axis: Hypothalamic–pituitary–adrenal axis
KOR: κ-Opioid receptor
LV: Lentiviral vector
mPFC: Medial prefrontal cortex
NAc: Nucleus accumbens
OX1R: Orexin 1 receptor
PARP-1: Poly [ADP-ribose] polymerase 1
PFC: Prefrontal cortex
POMC: Proopiomelanocortin
USV: Ultrasonic vocalization
VTA: Ventral tegmental area
miRNA: MicroRNA
