# Supplementary material for: Molecular pattern of a decrease in the rewarding effect of cocaine after an escalating-dose drug regimen
Source: Pharmacol Rep. 2022 Dec 31;75(1):85–98. doi: 10.1007/s43440-022-00443-3 (PMC9889529; doi:10.1007/s43440-022-00443-3)
Supplement: Supplementary file 1 — Supplementary file1 (DOC 29 kb) [file 43440_2022_443_MOESM1_ESM.doc]

| **Assay Name** | **miRBase ID** | **miRBase Accession Numbers** | **Mature miRNA Sequence** |
| --- | --- | --- | --- |
| mmu-miR-124a  hsa-miR-137 | mmu-miR-124-3p  hsa-miR-137-3p | MIMAT0000134  MIMAT0000429 | UAAGGCACGCGGUGAAUGCC  UUAUUGCUUAAGAAUACGCGUAG |

**Tab. 1.** TaqMan^TM^ MicroRNA Assays: mmu-miR-124a, hsa-miR-137
